# Immunological non-inferiority of a new fully liquid presentation of the MenACWY-CRM vaccine to the licensed vaccine: results from a randomized, controlled, observer-blind study in adolescents and young adults

**DOI:** 10.1080/21645515.2021.1981085

**Published:** 2021-10-06

**Authors:** Javier Díez-Domingo, Juan Carlos Tinoco, Airi Poder, Ener Cagri Dinleyici, Haylene Nell, Ignacio Salamanca de la Cueva, Tolga Ince, Edson Duarte Moreira, Khatija Ahmed, Kleber Luz, Yulia Kovshirina, Carlos Eduardo Medina Pech, Tauseefullah Akhund, Valerio Romolini, Marco Costantini, Thembile Mzolo, Barry Kunnel, Isabelle Lechevin, Marianna Aggravi, Paola Tiberi, K Narendran, Juan-Antonio García-Martínez, Venere Basile, Elena Fragapane, Maria Lattanzi, Michele Pellegrini

**Affiliations:** a Vaccine Research Area, FISABIO-Public Health, Valencia, Spain; b Infectious Disease, Hospital General de Durango, Durango, Mexico; c Kliiniliste Uuringute Keskus, Tartu, Estonia; d Pediatrics, Faculty of Medicine, Eskisehir Osmangazi University, Eskisehir, Turkey; e Tiervlei Trial Centre, Karl Bremer Hospital, Bellville, South Africa; f Instituto Hispalense de Pediatria, Seville, Spain; g Faculty of Medicine, Dokuz Eylul University, Izmir, Turkey; h Associação Obras Sociais Irmã Dulce and Oswaldo Cruz Foundation, Brazilian Ministry of Health, Salvador, Brazil; i Setshaba Research Centre, Tshwane, and Faculty of Health Sciences, Department of Medical Microbiology, University of Pretoria, Pretoria, South Africa; j Centro de Pesquisas Clinicas de Natal, Rio Grande do Norta, Brazil; k Infectious Diseases and Epidemiology, Siberian State Medical University, Tomsk, Russian Federation; l Medical Care and Research SA de CV, Mérida, Mexico; m Clinical Research and Development Centre, GSK, Siena, Italy; n Biostatistics, GSK, Siena, Italy; o Biostatistics, GSK, Amsterdam, The Netherlands; p Data Strategy & Management, Global Clinical Operations Development – R&D, GSK, Amsterdam, The Netherlands; q Clinical Laboratory Sciences, GSK, Rixensart, Belgium; r Technical Development, GSK, Siena, Italy; s Safety Evaluation and Risk Management, GSK, Siena, Italy; t Global Clinical Operations, GSK, Bangalore, India; u Medical Department, GSK, Madrid, Spain; v Global Clinical Delivery, Global Clinical Operations Development, GSK, Siena, Italy

**Keywords:** MenACWY-CRM, meningococcal serogroup A, human serum bactericidal assay, non-inferiority, adolescents

## Abstract

A fully liquid MenACWY-CRM vaccine presentation has been developed, modifying the meningococcal serogroup A (MenA) component from lyophilized to liquid. The safety and immunogenicity of the liquid presentation at the end of the intended shelf-life (aged for 24 or 30 months) were compared to the licensed lyophilized/liquid presentation. This multicenter, randomized (1:1), observer-blind, phase 2b study (NCT03433482) enrolled adolescents and young adults (age 10–40 years). In part 1, 844 participants received one dose of liquid presentation stored for approximately 24 months or licensed presentation. In part 2, 846 participants received one dose of liquid presentation stored for approximately 30 months or licensed presentation. After storage, the MenA free saccharide (FS) level was approximately 25% and O-acetylation was approximately 45%. The primary objective was to demonstrate non-inferiority of the liquid presentation to licensed presentation, as measured by human serum bactericidal assay (hSBA) geometric mean titers (GMTs) against MenA, 1-month post-vaccination. Immune responses against each vaccine serogroup were similar between groups. Between-group ratios of hSBA GMTs for MenA were 1.21 (part 1) and 1.11 (part 2), with two-sided 95% confidence interval lower limits (0.94 and 0.87, respectively) greater than the prespecified non-inferiority margin (0.5), thus meeting the primary study objective. No safety concerns were identified. Despite reduced O-acetylation of MenA and increased FS content, serogroup-specific immune responses induced by the fully liquid presentation were similar to those induced by the licensed MenACWY-CRM vaccine, with non-inferior anti-MenA responses. The safety profiles of the vaccine presentations were similar.

## Introduction

The incidence of invasive meningococcal disease (IMD), caused by *Neisseria meningitidis*, is highest among infants, adolescents, and young adults,^1^ with case fatality rates in these groups around 9–15% despite appropriate treatment.^2^ Most IMD cases are caused by one of six meningococcal serotypes (A, B, C, W, X, and Y).^3^

A quadrivalent meningococcal glycoconjugate vaccine that uses CRM_197_ as carrier protein (MenACWY-CRM; *Menveo*, GSK) is available in more than 60 countries. *Menveo* is approved for the active immunization of individuals at risk of exposure to meningococcal serogroups A, C, W, and Y to prevent IMD, and its safety and immunogenicity profiles are well established.^4^

The licensed *Menveo* presentation is prepared by reconstituting the lyophilized serogroup A (MenA-CRM_197_) component with the liquid serogroups C, W, and Y (MenCWY-CRM_197_) component just before injection. To simplify the vaccine administration process, avoid reconstitution errors, and save storage space, a fully liquid presentation of MenACWY-CRM has been developed that can be stored as a single vial. The structure of MenA conjugated polysaccharide is labile in aqueous solution,^5,6^ leading to an increased level of free saccharide (FS) and decreased O-acetylation of the MenA moiety over time.^7^ Preclinical studies found immunogenicity was reduced with the removal of O-acetyl groups from MenA,^8^ while a controlled clinical study of 1170 adults administered MenACWY-tetanus toxoid conjugate vaccine with decreased MenA O-acetylation showed no impact on vaccine immunogenicity.^9^

**Figure 1.**
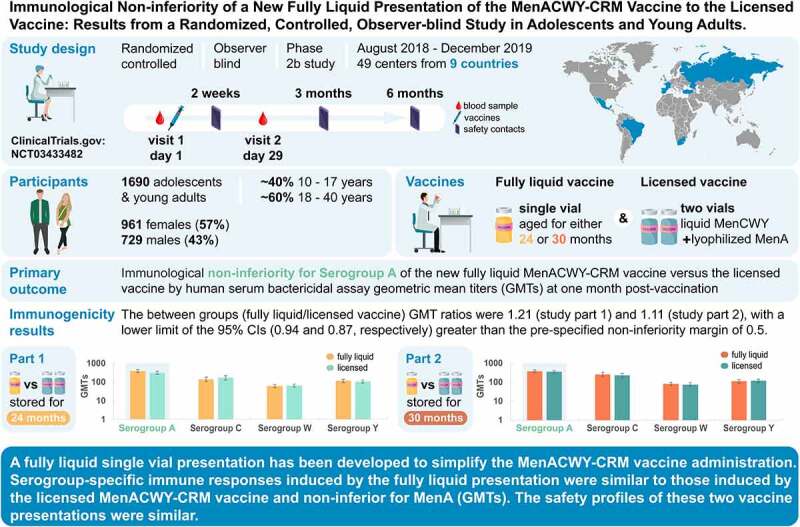
Study highlights.

In the present study, we examined if, by modifying the MenA component from lyophilized to liquid, subsequent controlled hydrolytic degradation with prolonged storage alters its immunogenicity in adolescents and young adults. We assessed the non-inferiority of the immune response against MenA induced by the investigational MenACWY-CRM liquid vaccine presentation at the end of its intended shelf-life (i.e., after storage at 2–8°C for approximately 24 and 30 months), as compared to the response induced by the licensed MenACWY-CRM vaccine. When this study was designed, the licensed vaccine form had a shelf-life of 24 months at the recommended storage temperature of 2–8°C.^10^ Immune responses against serogroups C, W, and Y, and the reactogenicity and safety of the study vaccines were also evaluated (Figure 1).

## Materials and methods

### Study design and participants

This randomized, controlled, observer-blind phase 2b study was conducted at 49 centers in nine countries (Brazil, Estonia, Finland, France, Mexico, Russia, South Africa, Spain, and Turkey) between August 30, 2018, and December 17, 2019 (ClinicalTrials.gov Identifier: NCT03433482). A study summary is available at www.gsk-studyregister.com (study identifier, 207467). The study was conducted in accordance with the Declaration of Helsinki and Good Clinical Practice and approved by the appropriate ethics committees. Before enrollment, all participants aged at least 18 years provided written informed consent and parents/legally acceptable representatives provided written informed consent for participants younger than 18 years.

Participants were healthy adolescents and adults aged 10–40 years. Complete inclusion and exclusion criteria are listed in the study protocol (https://www.gsk-studyregister.com/en/trial-details/?id=207467#documents-section). The study was conducted in two parts, with staggered timing. In part 1, participants received one dose of either the investigational MenACWY-CRM liquid presentation that had been stored at 2–8°C for approximately 24 months (ACWY_Liq24 group) or the licensed MenACWY-CRM presentation (*Menveo*; ACWY_1 group). In part 2, participants received one dose of the investigational MenACWY-CRM liquid presentation that had been stored at 2–8°C for approximately 30 months (ACWY_Liq30 group) or the licensed MenACWY-CRM presentation (*Menveo*; ACWY_2 group). For each study part, there were two visits (at vaccination and approximately 1 month afterward), and three telephone calls to each participant approximately 2 weeks, 3 months, and 6 months after vaccination.

Participants were enrolled and randomized in a 1:1 ratio for each study part. Allocation to a study group at each site was conducted via a central randomization system using a minimization procedure accounting for center, with stratification to ensure approximately 40% of participants were aged 10–17 years and 60% were aged 18–40 years. Due to differences in the presentation of the study vaccines, the study was observer blinded, i.e., vaccine recipients and those responsible for evaluating study endpoints were unaware of which vaccine was administered, while each vaccine was prepared and administered by authorized medical personnel who did not participate in the study’s clinical evaluations or assays.

The primary study objective was to demonstrate non-inferiority of the fully liquid MenACWY-CRM presentation stored for approximately 24 or 30 months to the licensed MenACWY-CRM presentation, as measured by human serum bactericidal assay (hSBA) geometric mean titers (GMTs) against MenA at 1-month post-vaccination. The primary outcome measure was the between-group ratio of hSBA GMTs at 1-month post-vaccination. The main secondary immunogenicity endpoints reported here are serogroup-specific hSBA GMTs post-vaccination, percentage of participants with hSBA titer ≥8 (considered as threshold for seroprotection^4^) pre- and post-vaccination, and percentage with a four-fold increase in hSBA titer post-vaccination. Other immunogenicity results, such as pre-vaccination hSBA GMTs, within-group GMT ratios, and percentage of participants with hSBA titer ≥ lower limit of quantitation can be found at ClinicalTrials.gov (https://www.clinicaltrials.gov/ct2/show/results/NCT03433482). Clinical tolerability and safety of the presentations were analyzed as secondary study endpoints; tolerability data other than adverse events (AEs), such as analgesic/antipyretic use, can be found at ClinicalTrials.gov.

### Study vaccine presentations

The MenACWY-CRM vaccine was administered intramuscularly in the deltoid region of the non-dominant arm (single 0.5 mL dose). The licensed presentation contains 10 µg of MenA-CRM_197_ and 5 µg each of serogroup C (MenC-CRM_197_), serogroup W (MenW-CRM_197_), and serogroup Y (MenY-CRM_197_) and is prepared by reconstituting the lyophilized MenA-CRM_197_ component (including, as excipients, sucrose and potassium dihydrogen phosphate) with the liquid MenCWY-CRM_197_ component (including, as excipients, sodium dihydrogen phosphate monohydrate, disodium phosphate dihydrate, sodium chloride, and water for injection) just before injection. The investigational liquid presentation contained the same CRM-conjugated meningococcal serogroup components as the licensed presentation. FS and O-acetylation levels of the liquid presentation MenA moiety were monitored via high-performance anion-exchange chromatography with pulsed amperometric detection and proton nuclear magnetic resonance, respectively. MenA FS levels were approximately 25% and O-acetylation was approximately 45% at the end of intended shelf-life of 30 months.

### Serological analyses

Two blood samples of 20 mL each were collected before vaccination and approximately 1-month post-vaccination. The induction of bactericidal antibodies against *N. meningitidis* serogroups A, C, W, and Y was determined by a validated hSBA performed by GSK, Clinical Laboratory Sciences, Wavre, Belgium.

### Safety analyses

Participants were observed at the study centers for 30 minutes after vaccination for immediate reactions. Solicited local (erythema, induration, and pain at the injection site) and systemic (arthralgia, chills, fatigue, headache, loss of appetite, myalgia, and nausea) AEs and body temperature were reported by participants on electronic diaries for 7 days following vaccination. The severity of solicited AEs (apart from erythema and induration) was classified as mild, moderate, or severe (preventing normal activity or, for loss of appetite, not eating at all, or, for erythema and induration, >100 mm in diameter). Fever was defined as body temperature ≥38°C.

Unsolicited AEs were recorded for up to 1-month post-vaccination. Serious AEs (SAEs), medically-attended AEs, and AEs leading to withdrawal were reported over the entire 6-month study period and the causal relationship of AEs to vaccination was assessed by study investigators.

### Statistical analysis

With 375 evaluable participants in each study group, there was approximately 90% power to reject the null hypothesis that the between-group ratio of hSBA GMTs against MenA for ACWY_Liq24 to ACWY_1 and ACWY_Liq30 to ACWY_2 was ≤0.5. Overall power for both hypotheses (part 1 and part 2) was approximately 81%. Anticipating a drop-out rate of 10%, enrollment of 1668 participants (417 per group) was planned.

The primary immunogenicity analyses were conducted on the per-protocol population for immunogenicity, defined as participants who received study vaccination correctly, had no major protocol deviations, and had assay results available for at least one serogroup. Non-inferiority of the investigational presentation to the licensed presentation was to be concluded if the lower limit of the two-sided 95% confidence interval (CI) for the between-groups ratio of adjusted hSBA GMTs against MenA was greater than 0.5 (non-inferiority margin) 1-month post-vaccination. GMTs adjusted for pre-vaccination titer were calculated using an analysis of covariance model with pre-vaccination titer as covariate and group and country as factors.

Analysis of all secondary immunogenicity objectives was descriptive. For each serogroup, percentages of participants with a four-fold increase in post-vaccination hSBA titer and percentages with hSBA titer ≥8 were analyzed, with two-sided 95% Clopper-Pearson CIs.^11^ Differences in percentages between groups were calculated using the Miettinen and Nurminen method.^12^ All participants who received study vaccination and provided safety data were included in the descriptive safety analyses.

Statistical analyses were performed using Statistical Analysis System Life Science Analytics Framework with SAS version 9.4 (SAS Institute Inc., Cary, NC, USA).

## Results

### Study population

**Figure 2.**
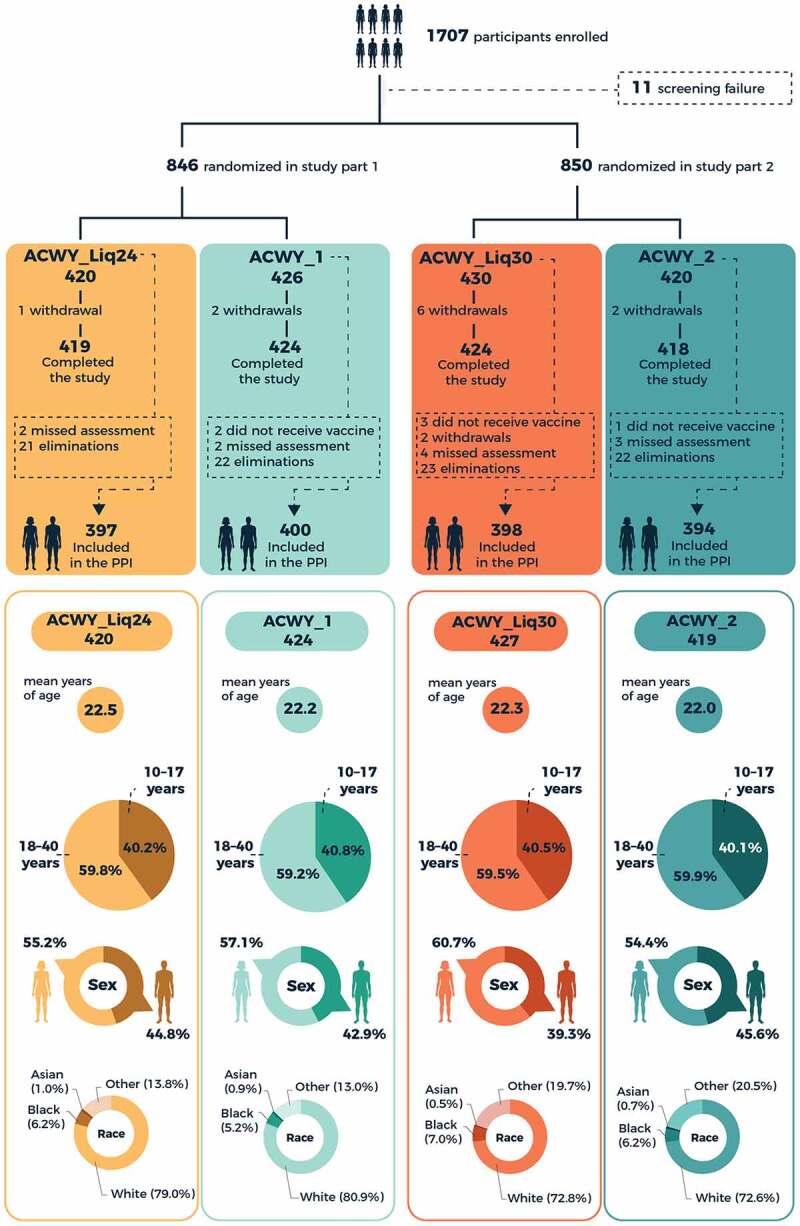
Trial profile and demographic characteristics of vaccinated study participants. PPI, per-protocol population for immunogenicity.

Of 1707 healthy adolescents and young adults aged 10–40 years enrolled in the study, 844 received the MenACWY-CRM liquid presentation stored for 24 months or licensed presentation (Part 1), and 846 received the liquid presentation stored for 30 months or licensed presentation (Part 2) (Figure 2). Numbers of participants included in the immunogenicity analyses are shown in Figure 2. Demographic characteristics were similar across the study groups (Figure 2). Approximately 40% of participants in each group were aged 10–17 years.

### Immunogenicity

At 1-month post-vaccination, MenA hSBA adjusted GMTs were 386.66 in the ACWY_Liq24 group versus 318.34 in the ACWY_1 group, and 387.06 in the ACWY_Liq30 group versus 348.89 in the ACWY_2 group (Table 1).

**Table 1.** Adjusted hSBA geometric mean titers (GMTs) 1-month post-vaccination and between-group ratios for groups that received MenACWY-CRM liquid vaccine aged for 24 or 30 months (ACWY_Liq24 and ACWY_Liq30) versus groups that received licensed MenACWY-CRM vaccine (ACWY_1 and ACWY_2) (per-protocol population for immunogenicity)

	ACWY_Liq24		ACWY_1		GMT ratio, ACWY_Liq24: ACWY_1 (95% CI)	ACWY_Liq30		ACWY_2		GMT ratio, ACWY_Liq30: ACWY_2 (95% CI)
	N	GMT (95% CI)	N	GMT (95% CI)		N	GMT (95% CI)	N	GMT (95% CI)	
Overall										
Serogroup A	363	386.66 (319.47–467.97)	373	318.34 (264.14–383.67)	1.21 (0.94–1.57)	356	387.06 (322.72–464.24)	349	348.89 (290.09–419.61)	1.11 (0.87–1.42)
Serogroup C	385	143.48 (110.49–186.30)	377	171.74 (131.74–223.87)	0.84 (0.58–1.19)	376	256.70 (195.29–337.41)	377	226.09 (171.62–297.85)	1.14 (0.79–1.64)
Serogroup W	372	62.63 (50.99–76.92)	388	66.37 (54.26–81.18)	0.94 (0.72–1.24)	374	83.23 (68.19–101.60)	366	75.42 (61.56–92.41)	1.10 (0.84–1.45)
Serogroup Y	379	115.66 (94.13–142.13)	390	106.47 (86.93–130.42)	1.09 (0.82–1.44)	386	112.38 (91.56–137.92)	377	117.56 (95.39–144.89)	0.96 (0.72–1.26)
Age 10–17 y										
Serogroup A	154	441.33 (338.41–575.54)	153	422.96 (325.99–548.77)	1.04 (0.74–1.47)	151	397.32 (303.93–519.41)	138	382.73 (288.50–507.73)	1.04 (0.73–1.48)
Serogroup C	159	201.13 (137.19–294.88)	155	243.20 (165.65–357.06)	0.83 (0.51–1.35)	157	286.66 (181.94–451.65)	153	366.56 (230.68–582.47)	0.78 (0.44–1.40)
Serogroup W	150	40.72 (29.84–55.55)	158	51.38 (38.14–69.21)	0.79 (0.53–1.18)	151	71.12 (50.98–99.23)	146	57.61 (40.85–81.24)	1.23 (0.81–1.88)
Serogroup Y	157	109.92 (80.31–150.46)	157	112.85 (82.99–153.44)	0.97 (0.65–1.46)	155	123.01 (87.98–171.98)	154	92.01 (65.47–129.30)	1.34 (0.87–2.06)
Age 18–40 y										
Serogroup A	209	362.33 276.85–474.18)	220	268.44 (206.90–348.30)	1.35 (0.94–1.94)	205	352.92 (273.22–455.86)	211	307.22 (238.98–394.94)	1.15 (0.82–1.61)
Serogroup C	226	106.05 (75.27–149.43)	222	125.38 (88.51–177.61)	0.85 (0.53–1.36)	219	221.98 (157.63–312.60)	224	156.43 (110.92–220.63)	1.42 (0.90–2.23)
Serogroup W	222	85.60 (64.97–112.79)	230	77.66 (59.13–102.00)	1.10 (0.76–1.61)	223	93.09 (71.32–121.52)	220	89.60 (68.42–117.33)	1.04 (0.73–1.48)
Serogroup Y	222	124.26 (94.11–164.08)	233	103.99 (79.17–136.58)	1.19 (0.82–1.75)	231	106.38 (81.03–139.65)	223	134.99 (102.20–178.30)	0.79 (0.55–1.13)

CI, confidence interval; GMT, geometric mean titer; N, number of participants who received vaccination and had assay results available; y, years of age.

The lower limit of the two-sided 95% CI for the between-groups adjusted GMT ratio against serogroup A was 0.94 for ACWY_Liq24 to ACWY_1 and 0.87 for ACWY_Liq30 to ACWY_2, both greater than the non-inferiority margin of 0.5, fulfilling the primary immunogenicity endpoints of the study.

Adjusted hSBA titers against serogroups C, W, and Y were similar with the aged liquid and licensed presentations (Table 1). Applying the same non-inferiority limit as used for MenA, non-inferiority would also be demonstrated for these serogroups, as the lower limit of the two-sided 95% CI for the between-groups adjusted GMT ratios ranged between 0.58 (MenC) and 0.84 (MenW) (Table 1). GMT results by age group (10–17 years and 18–40 years) were comparable with the aged liquid and licensed presentations (Table 1).

**Figure 3.**
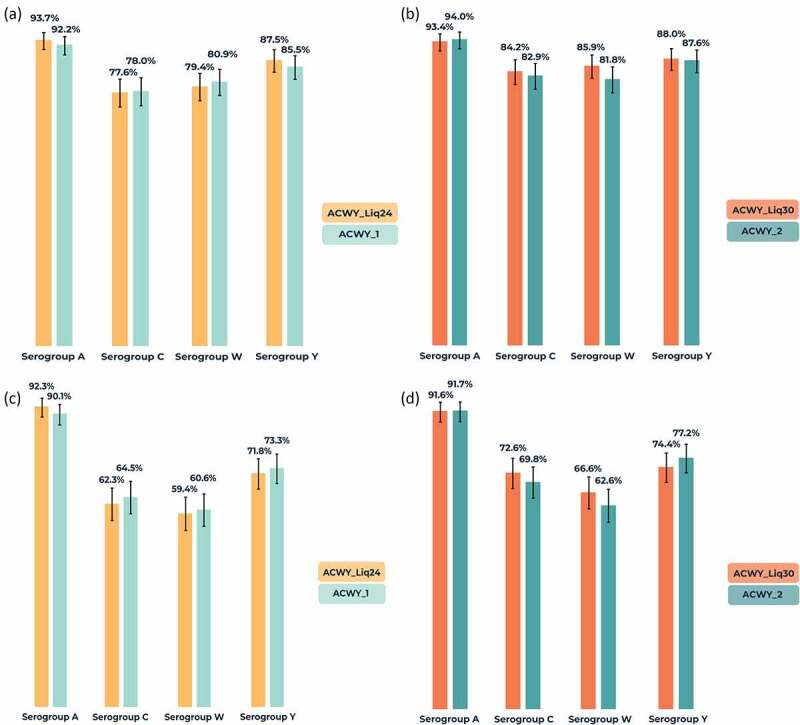
Percentages of participants (with 95% confidence intervals) with (a,b) human serum bactericidal assay (hSBA) titer ≥8 at 1-month post-vaccination and (c,d) four-fold increase in hSBA titer from baseline to 1-month post-vaccination against serogroups A, C, W, and Y (per-protocol population for immunogenicity).

Baseline percentages of participants with hSBA titer ≥8 varied according to serogroup and were lowest in both study parts for MenA (10.3–13.5%), 41.5–50.6% for MenC, 28.5–31.7% for MenW, and 21.5–22.8% for MenY. For all serogroups, 1-month seroprotection rates (hSBA titer ≥8) were similar in the liquid and licensed presentation comparisons, ranging from 77.6% (MenC) to 93.7% (MenA) in the ACWY_Liq24 group, from 84.2% (MenC) to 93.4% (MenA) in the ACWY_Liq30 group, and from 78.0% (MenC) to 94.0% (MenA) with the licensed presentation (Figure 3a,b). Results by presentation were also similar in each age group category (data not shown). Percentages with hSBA titer ≥8 in the 10–17 years group ranged from 79.0% (MenC) to 95.9% (MenA) and, in the 18–40 years group from 75.1% (MenC) to 92.8% (MenA). Percentages of participants with a four-fold increase in antibody GMTs versus baseline were also similar in the presentation comparisons and highest for MenA (Figure 3c,d). Percentages with a four-fold increase from baseline in the 10–17 years group ranged from 64.0% (MenW) to 95.5% (MenA) and, in the 18–40 years group, from 53.5% (MenW) to 90.1% (MenA).

Four-fold increase in hSBA titer post-vaccination defined as four times the limit of detection (LOD) or the lower limit of quantitation (LLOQ) (whichever was greater) for individuals with pre-vaccination titers < LOD, four times the LLOQ for individuals with pre-vaccination titers between LOD and LLOQ (inclusive), or four times the pre-vaccination titer for individuals with pre-vaccination titers > LLOQ. LLOQ was 5 for MenA, 6 for MenC, 7 for MenW, and 6 for MenY, and LOD was 4 for each serogroup. ACWY_Liq24 group received investigational MenACWY-CRM liquid vaccine aged for 24 months; ACWY_Liq30 group received liquid vaccine aged for 30 months; ACWY_1 and ACWY_2 groups received licensed MenACWY-CRM vaccine. Number of participants with data available on hSBA titer ≥8 (serogroups A, C, W, Y, respectively): 378, 388, 389, 384 for ACWY_Liq24 group; 384, 382, 392, 392 for ACWY_1 group; 377, 379, 389, 393 for ACWY_Liq30 group; 367, 379, 384, 386 for ACWY_2 group. Number of participants with data available on four-fold increase in hSBA titer (serogroups A, C, W, Y, respectively): 363, 385, 372, 379 for ACWY_Liq24 group; 373, 377, 388, 390 for ACWY_1 group; 356, 376, 374, 386 for ACWY_Liq30 group; 349, 377, 366, 377 for ACWY_2 group. Percentage in ACWY_Liq24 and ACWY_1 groups with hSBA titer ≥8 at 1-month post-vaccination. Percentage in ACWY_Liq30 and ACWY_2 groups with hSBA titer ≥8 at 1-month post-vaccination. Percentage in ACWY_Liq24 and ACWY_1 group with four-fold increase in hSBA titer from baseline to 1-month post-vaccination. Percentage in ACWY_Liq30 and ACWY_2 groups with four-fold increase in hSBA titer from baseline to 1-month post-vaccination.

### Reactogenicity and safety

**Figure 4.**
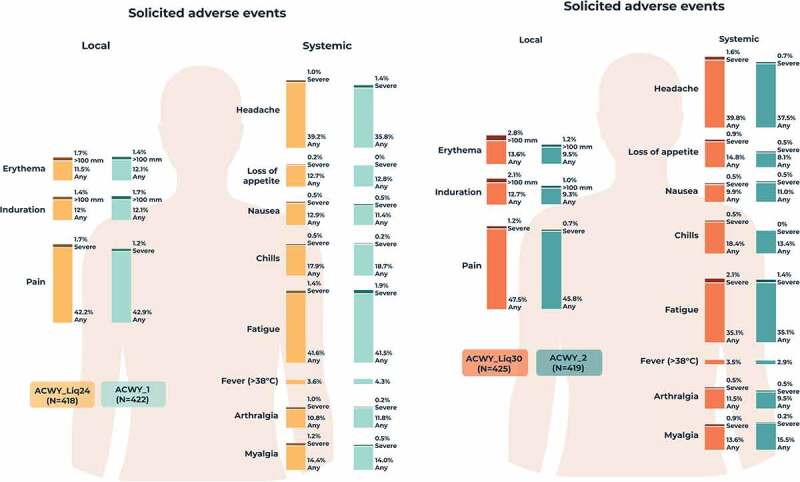
Percentages of participants reporting solicited local and systemic adverse events within 7 days of vaccination (solicited safety population).

The percentage of participants reporting at least one solicited local AE during the 7-day post-vaccination period was 47.4% in the ACWY_Liq24 group, 45.7% in the ACWY_1 group, 49.6% in the ACWY_Liq30 group, and 47.5% in the ACWY_2 group. The most commonly reported solicited local AE was pain at the injection site in all groups (Figure 4). Solicited systemic AEs were reported by 60.5% of participants in the ACWY_Liq24 group, 57.8% in the ACWY_1 group, 57.6% in the ACWY_Liq30 group, and 55.4% in the ACWY_2 group. The most commonly reported solicited systemic AEs were headache and fatigue in each group (Figure 4). Solicited local and systemic AEs were generally mild to moderate in intensity, with few reports of severe solicited AEs (Figure 4) and resolved within 7 days of vaccination. There were no reports of fever with temperature ≥40°C in the ACWY_Liq24 group and ACWY_1 group, and one report in both the ACWY_Liq30 group and ACWY_2 group, occurring within 3 days of vaccination.

Severe defined as preventing normal activity or, for loss of appetite, not eating at all. ACWY_Liq24 group received investigational MenACWY-CRM liquid vaccine aged for 24 months; ACWY_Liq30 group received liquid vaccine aged for 30 months; ACWY_1 and ACWY_2 groups received licensed MenACWY-CRM vaccine. N, number of participants.

**Figure 5.**
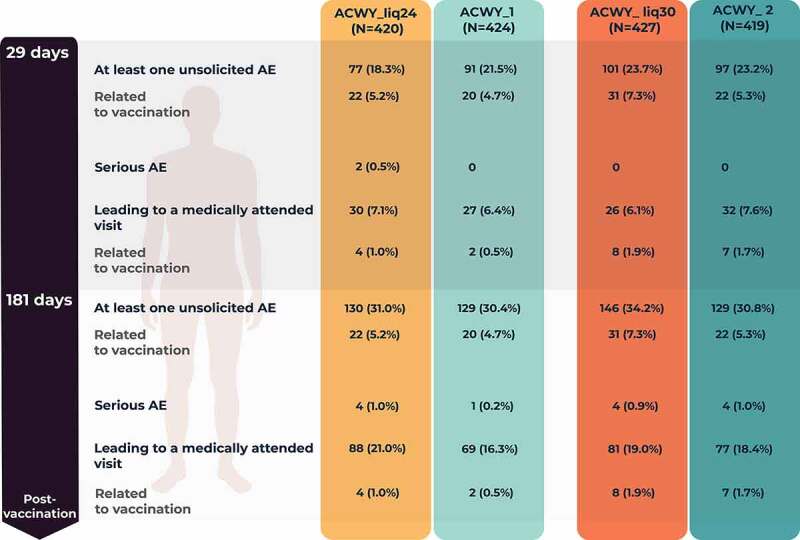
Number (percentage) of participants reporting unsolicited adverse events (AEs) during the 1-month and 6-month post-vaccination periods (unsolicited safety population).

Percentages of participants reporting unsolicited AEs within 1 month or 6 months after vaccination were similar among the four study groups (Figure 5). The most frequently reported unsolicited AEs within 1 month after vaccination were headache in the ACWY_Liq24 (3.1%), ACWY_Liq30 (6.1%), and ACWY_2 (5.7%) groups, and nasopharyngitis (3.5%) in the ACWY_1 group. In the 1-month post-vaccination period, the most frequently reported unsolicited AE considered related to vaccination was headache (1.2%) in the ACWY_Liq24 group and ACWY_1 group; injection site pain (2.8%), erythema (2.3%), induration (1.9%), and headache (1.6%) in the ACWY_Liq30 group; and, in the ACWY_2 group, injection site pain (2.1%), erythema (1.7%), and headache (1.7%).

ACWY_Liq24 group received investigational MenACWY-CRM liquid vaccine aged for 24 months; ACWY_Liq30 group received liquid vaccine aged for 30 months; ACWY_1 and ACWY_2 groups received licensed MenACWY-CRM vaccine. N, number of participants.

A similar percentage of participants in the four study groups reported a medically attended unsolicited AE during the study (Figure 5). In 6 months, four SAEs (tooth abscess, soft tissue injury, non-infective appendicitis, and ruptured ovarian cyst) were reported by four participants in the ACWY_Liq24 group, one SAE (malignant melanoma) was reported in the ACWY_1 group, four SAEs (post-procedural hemorrhage, tibia fracture, spontaneous abortion, and depression) were reported by four participants in the ACWY_Liq30 group, and four SAEs (phimosis, otitis externa, tension headache, and adnexa uteri pain) were reported by four participants in the ACWY_2 group. None of the SAEs were assessed as related to study vaccination. No participant was withdrawn from the study due to an AE and no deaths were reported.

## Discussion

A fully liquid single vial presentation has been developed to simplify administration of the MenACWY-CRM vaccine. Since the structure of MenA undergoes some degradation over time in aqueous solution,^5,6^ we assessed if there was an impact on the immunogenicity of MenA in the fully liquid presentation following controlled degradation during storage at 2–8°C for approximately 24 and 30 months, as indicated by FS generation and reduced O-acetylation of the MenA moiety. The participants were adolescents and young adults, with around 40% aged 10–17 years, and thus representative of a main target group for immunization to prevent IMD outbreaks.^3^

This study successfully demonstrated the immunological non-inferiority of the MenACWY-CRM liquid presentation after 24 and 30 months’ storage versus the licensed MenACWY-CRM presentation against MenA. The immunogenicity of the MenACWY-CRM liquid presentation at the end of intended shelf-life was similar to *Menveo* when analyzed by meningococcal serogroup according to different immunogenicity endpoints, and the clinical tolerability and safety profiles of the two vaccine presentations were comparable. No safety concerns were identified, and the overall reactogenicity and safety profile of the fully liquid presentation was consistent with the safety profile of the licensed MenACWY-CRM vaccine, as reported in clinical development studies in adolescents and adults.^4,10^

Since only the MenA component was modified from lyophilized to liquid in the investigational presentation, it was not planned to formally assess the non-inferiority of immune responses against serogroups CWY, which were unchanged. Nevertheless, applying the same non-inferiority criterion as pre-selected for MenA, immunological non-inferiority would also be demonstrated for these three serogroups. Analyses by age group (10–17 years and 18–40 years) also showed similar immune responses against each serogroup with the aged fully liquid presentation versus the licensed presentation. Our study therefore provides evidence that, despite findings from pre-clinical models,^8^ de-O-acetylation of MenA and an increased FS content, up to the levels measured in this study, do not have an impact on the MenACWY-CRM vaccine’s ability to induce immune responses in adolescents and young adults. This evidence confirms clinical results obtained with a MenACWY-tetanus toxoid conjugate vaccine with different degrees of MenA de-O acetylation, which also showed no impact on vaccine immunogenicity.^9^

Notable proportions of the adolescents and young adults included in this study (up to 51% for MenC) had hSBA titers ≥8 against serogroups CWY before vaccination, presumably resulting from natural exposure to *N. meningitidis*. This is supported by epidemiological observations from the regions involved in our study, which also show infrequent outbreaks due to MenA.^3^ The lower baseline proportion of participants with putative protective titers against MenA is likely to have contributed to the higher proportion achieving a four-fold increase in titers for MenA post-vaccination than for serogroups CWY.

The hSBA GMTs against MenA induced by the liquid presentation were over 300 in each study group, much higher than hSBA GMTs against MenA reported in previous MenACWY-CRM vaccine clinical trials in adolescents and adults.^13–17^ This is likely to be due to the new validated serological hSBA adopted for this study, which is an agar-overlay assay with a higher throughput than the manual tilt hSBA used during *Menveo* clinical development and which includes a different MenA indicator strain (3125).^18^ The 3125 strain was selected over the former F8238 MenA antigen to allow bactericidal antibodies to be detected against most invasive MenA strains expressing the L(3,7)9 or L10 immunotype, rather than against a strain usually observed to be involved in carriage, such as F8238.

While interpretation of the overall results of this study is partially limited by a lack of formal non-inferiority assessments for serogroups C, W, and Y, these components were not modified in the investigational liquid presentation. The descriptive analyses indicate similar immunogenicity results for all serogroups with both presentations, irrespective of the immunogenicity endpoint evaluated.

In conclusion, immune responses for all vaccine meningococcal serogroups with the aged fully liquid MenACWY-CRM presentation were similar to those elicited by the licensed vaccine and were non-inferior against MenA, the only component modified in the liquid presentation. No safety concerns arose following its administration and the reactogenicity and safety profiles of the two vaccine presentations were similar. Our data provide further clinical evidence of no impact on vaccine immunogenicity of an increased MenA FS content and reduced O-acetylation, to the percentages monitored in this study, in healthy adolescents and adults. The results from this randomized, multicenter, controlled study support the introduction of the improved fully liquid presentation of MenACWY-CRM vaccine, once approved.

## Additional information

*Menveo* is a trademark owned by or licensed to the GSK group of companies.

All authors participated in the design or implementation of the study and were involved in the analysis and interpretation of the results and the development of this article. All authors had full access to the data and gave final approval before submission.

Anonymized individual participant data and study documents can be requested for further research from www.clinicalstudydatarequest.com.
